# The Use of Three-Dimensional Printing Model in the Training of Choledochoscopy Techniques

**DOI:** 10.1007/s00268-018-4731-6

**Published:** 2018-07-31

**Authors:** Ang Li, Rui Tang, Zhixia Rong, Jianping Zeng, Canhong Xiang, Lihan Yu, Wenping Zhao, Jiahong Dong

**Affiliations:** 1 Department of Hepatopancreatobiliary Surgery, Tsinghua University Affiliated Beijing Tsinghua Changgung Hospital, No. 168 Litang Road, Changping District, Beijing, 102218 China; 2 Service of Hepatobiliary and Pancreatic Surgery and Liver Transplantation, Centre Hospitalier de l’Université de Montréal (CHUM), Université de Montréal, Montréal, QC Canada

## Abstract

**Aim:**

To evaluate the application value of a three-dimensional (3D) printing model in the training of choledochoscopy techniques.

**Materials and methods:**

Imaging data from two patients with biliary dilatation were used to produce two 3D reconstruction models which were subsequently constructed into 3D printing models (No. 1 and No. 2). Four hepatobiliary surgeons evaluated the anatomical accuracy and academic teaching value of the printed models. Twenty resident trainees with no prior experience in any kind of endoscopic techniques were randomly and symmetrically divided into two groups. The training group (A) used the 3D model No. 1 in the learning of biliary tract anatomy and practice techniques of choledochoscopy. The control group (B) got the virtual 3D image of the same model on computer for learning. After 4 weeks, the model No. 2 was used to reassess the trainees’ subjective and objective progress in anatomy familiarity and choledochoscopy manipulations.

**Results:**

All consulted surgeons agreed that the 3D models realistically reproduced the anatomy of the biliary system. All trainees in group A agreed or strongly agreed that the 3D models provided good anatomical realism, enhanced their experience in the training of choledochoscopy techniques, and aided in their learning of biliary anatomy. With the practice went on, they increased the accuracy and showed a reduction in operation time on the model No. 1. During final examination with model No. 2, the rate of correct anatomical structure identification in training group was significantly higher than group B (*p *< 0.05).

**Conclusion:**

The 3D printed biliary tract model is an excellent teaching tool in the training of choledochoscopy techniques. The 3D model is anatomically realistic and can improve the trainee’s anatomical knowledge and endoscopic skills.

## Introduction

Three-dimensional (3D) reconstruction and three-dimensional printing (3DP) have numerous applications in modern medicine, and play an increasingly important role in medical and surgical education [1]. 3D visualization of anatomical structures provides a direct sensory experience which reduces the need to rely on recall and imagination. The anatomy of the biliary system, with its tree-like ductal structures that burrow deep into the hepatic parenchyma, is particularly complex and difficult to master, especially for resident trainees. 3D reconstructions have previously been applied in hepatobiliary surgery with favorable results, aiding the surgeon in the spatial recognition of biliary branches, allowing surgical procedures to be carried out with more precision [2].

Choledochoscopy is an important method of diagnosis and treatment for biliary tract disease. Due to the complexity of bile ducts, even an experienced endoscopist may disorient him or herself, leading to anatomical confusion and leading to consequences such as missed lesions, ultimately affecting the efficiency and outcome of the procedure [3].

Reports on the use of 3D printed models for the training of rigid bronchoscopy are available in the literature; however, experience in application of 3D models in the training of flexible endoscopy is scarce [4]. Since flexible scopes are more difficult to manipulate than rigid instruments, mastering flexible choledochoscopy requires longer training times to consolidate anatomical knowledge and dexterity. We hereby evaluate a novel application of 3D printed biliary tract models and assess its academic value in the learning of choledochoscopy techniques.

## Materials and methods

**Fig. 1:**
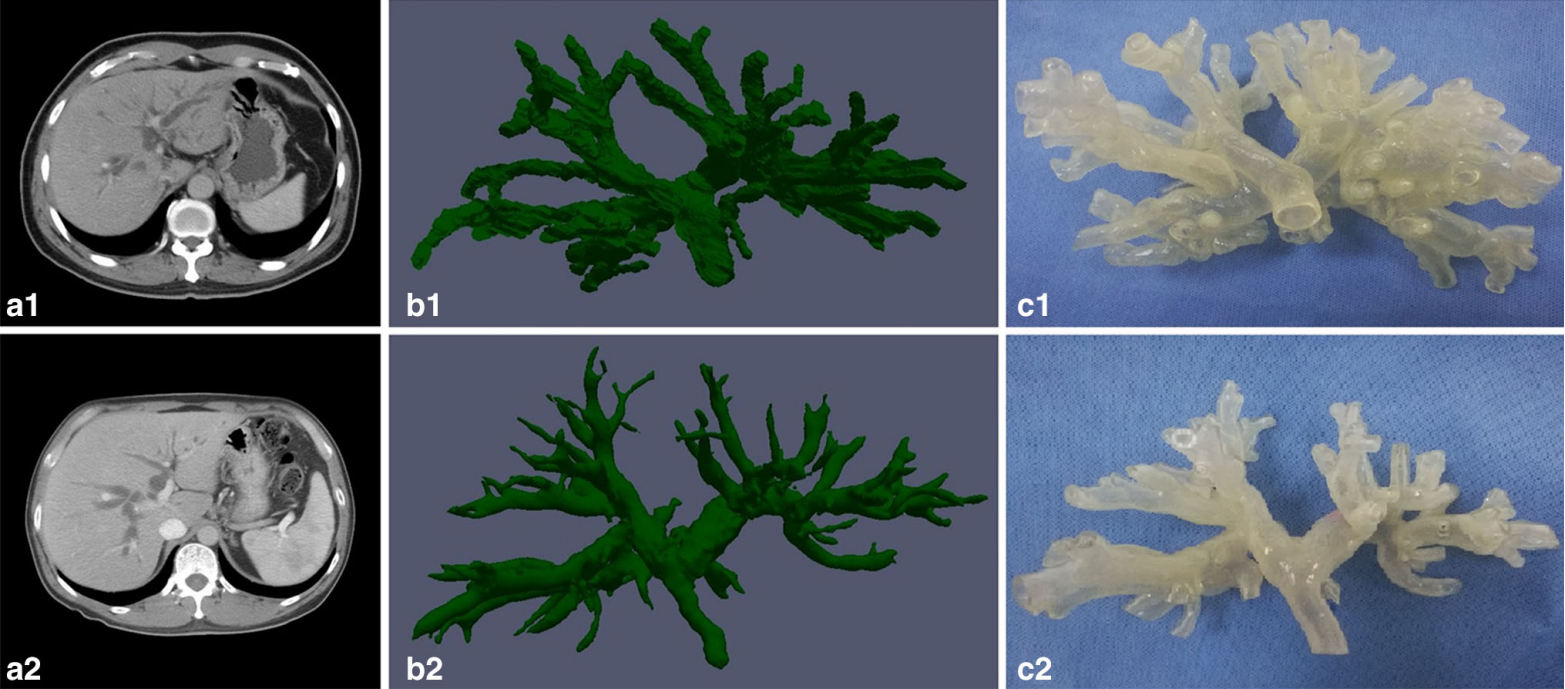
Computed tomography (CT) images (**a1**, **a2**), three-dimensional (3D) reconstruction (**b1**, **b2**) and three-dimensional printing (3DP) (**c1** Model No. 1, **c2** Model No. 2) of two patients with biliary tract dilatation

Computed tomography (CT) images from two patients with biliary tract dilatation were obtained and coded into the Digital Imaging and Communications in Medicine (DICOM) format. The image files were then processed by a 3D rendering software (Hisense Computer Assisted Surgery System, Hisense, Qingdao, China) for 3D reconstruction. 3DP was then performed with a 3D printer (Stratasys Connex3350, USA) and hollow-centered bile duct reconstruction models were produced on a 1:1 scale (Fig. 1).

Four hepatobiliary surgeons from the Beijing Tsinghua Changgung Hospital were consulted and each completed a feedback questionnaire to evaluate the value of the 3DP biliary tract models as an anatomical teaching tool on a 5-point Likert scale (1 = strongly disagree; 2 = disagree; 3 = neither agree nor disagree; 4 = agree; and 5 = strongly agree). Anatomical realism and application value as a teaching aid were assessed. Anatomical realism evaluation was based on the spatial structures of bile ducts of segments II, III and the opening of IV from left liver while segments VI, VII and ventral and dorsal subsegment of VIII from right liver.

Twenty junior resident trainees in hepatobiliary surgery from the Beijing Tsinghua Changgung Hospital with no prior experience in the manipulation of any kind of endoscope were enrolled as study participants. They were randomly divided into two groups by computer with 10 people for each. Group A is the training group, while group B is the control group. Bile duct endoscopy examinations in this study were performed with a CHF-V choledochoscopy (CHF-V, Olympus Corporation, Tokyo, Japan). Before training project started, all trainees observed real clinical choledochoscopic examination twice in 1 week and were taught the basic skills of choledochoscopy.

The trainees in group A were first submitted to a pretest using the 3DP models No. 1 inside a dark box, and their ability to correctly identify bile ducts from the left, right, right anterior, right posterior, B IV (bile duct of segment IV) and B VIII was evaluated. The same model was then used to familiarize the group A with biliary tract anatomy and practice choledochoscopy manipulations. Meanwhile group B just got the virtual 3D images of the same model on computer for learning.

**Fig. 2:**
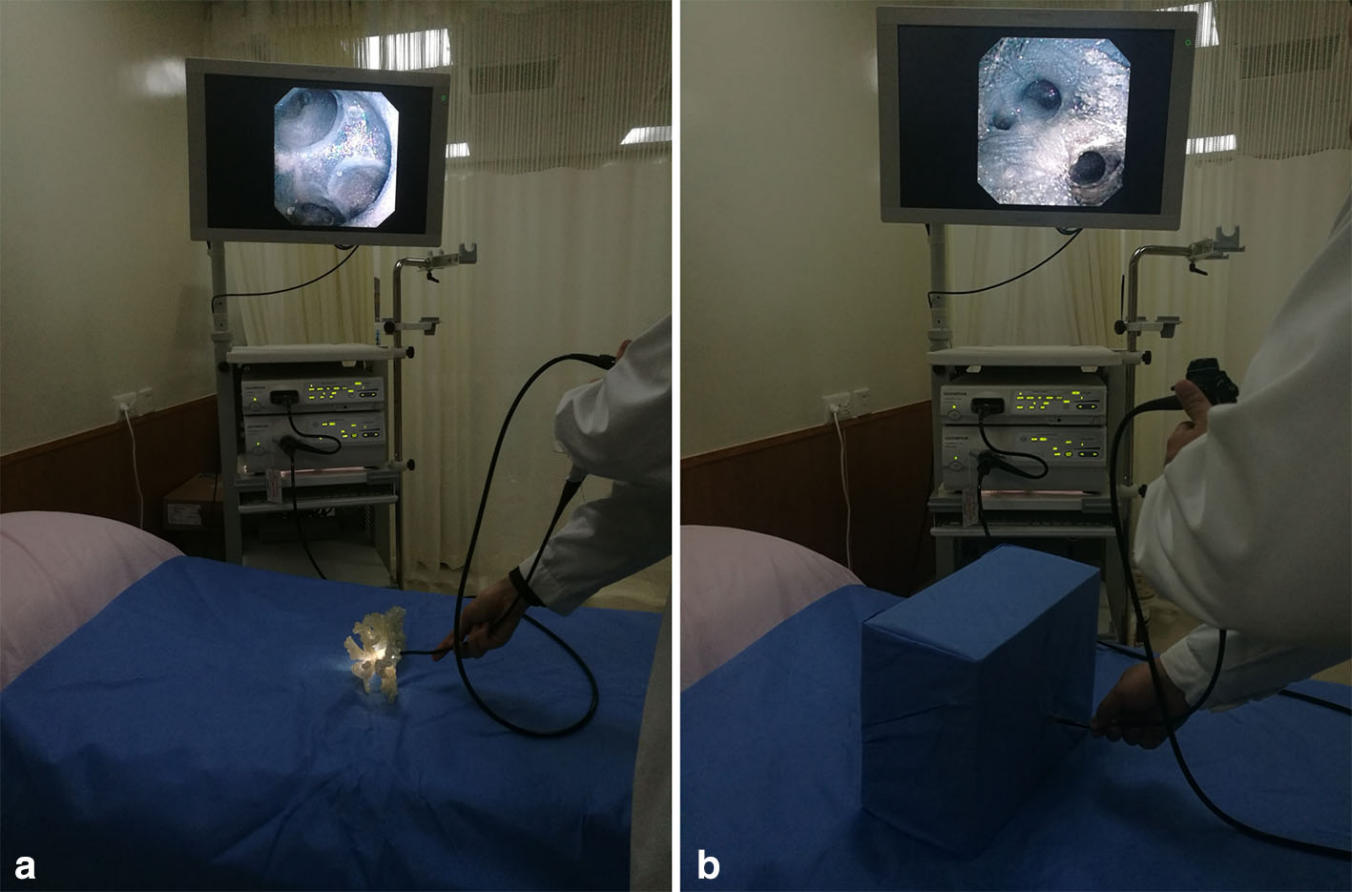
Choledochoscopy techniques practice training (**a**) and test (**b**) for junior resident trainees

The training of group A lasted for 4 weeks and twice in every week. At the end of every 2 weeks, these trainees were re-tested using model No. 1 inside a dark box (examination 1, examination 2). Their accuracy and operation time were recorded. Four weeks later, the trainee residents from both groups were submitted to a posttest finally without prior notification, and the same anatomical knowledge and choledochoscopic skills were evaluated on model No. 2 (Fig. 2). The virtual 3D image of model No. 2 was sent to all twenty trainees one hour before the final examination. Results from the two groups were compared. Two hepatobiliary surgeons from the Beijing Tsinghua Changgung Hospital served as examiners for both evaluations. Examination results were then compared with the Chi-squared test and Mann–Whitney *U* test using IBM SPSS Statistics 22 (IBM Corporation, Armonk, NY, USA). A *p* value of <0.05 was considered statistically significant, and very significant if <0.01. The trainees of group A then subjectively evaluated their learning process on a 5-point Likert scale.

## Results

All four hepatobiliary surgeons consulted agreed or strongly agreed that the 3D models were anatomically realistic and accurately reproduced biliary tract structures and helped for verisimilitude medical practice (Table 1).

**Table 1:** Five-point Likert scale for experts and students of medical practice teaching

	Experts (*n *= 4)					Students in group A (*n *= 10)				
	Realism of anatomical illustration					Realism of anatomical illustration				
Likert scale	1	2	3	4	5	1	2	3	4	5
Number	0	0	0	3	1	0	0	0	4	6
	Verisimilitude medical practice					Promoting examination practice				
Likert scale	1	2	3	4	5	1	2	3	4	5
Number	0	0	0	2	2	0	0	0	3	7

All resident trainees in group A also agreed or strongly agreed that the 3D models provided anatomical realism and favorably enhanced their learning experience in the techniques of choledochoscopy (Table 1).

**Fig. 3:**
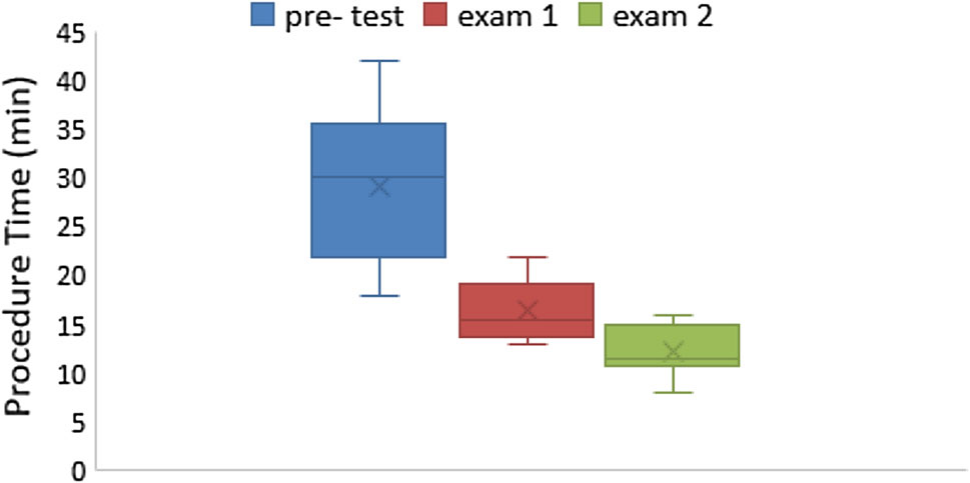
As the practice moved on, choledochoscopic examination takes less and less time in group A

**Fig. 4:**
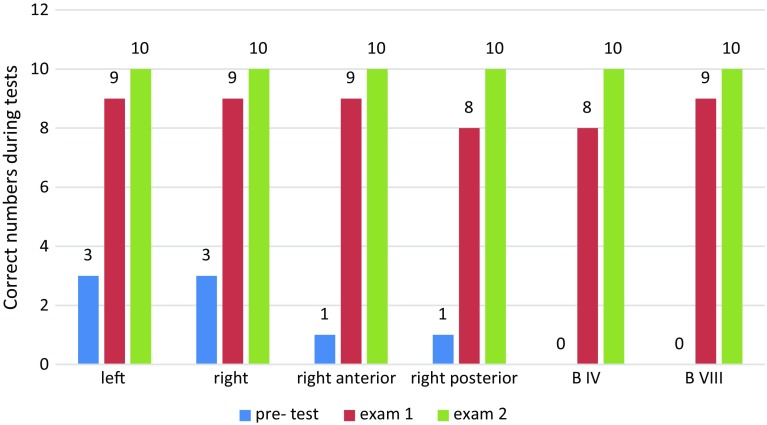
After training, the rate of correct anatomical identification of biliary tract structures in group A gradually increased

**Fig. 5:**
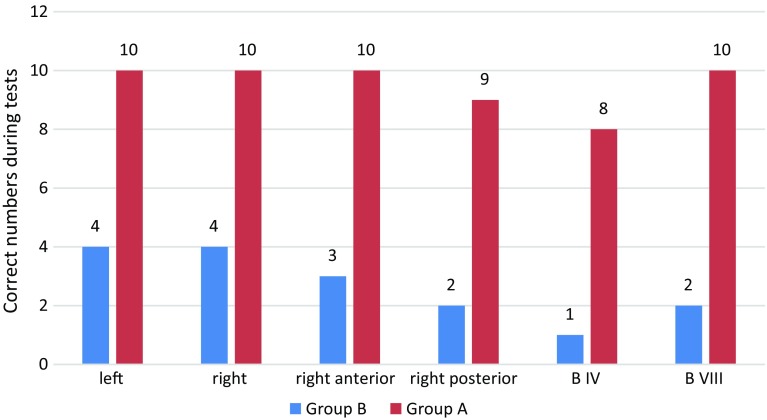
The posttest showed the accuracy is significantly higher in group A than group B

After training, there was significant improvement in the group A trainees’ evaluation results, and the rate of correct anatomical identification of biliary tract structures in model No. 1 also increased significantly with reduction in operation time (Table 2, Figs. 3 and 4). Significant improvement occurred in the first 2 weeks; meanwhile, in the second 2 weeks accuracy improved but not significantly and operation time reduced significantly during both first and second 2 weeks. During final examination on model No. 2, training group showed much better accuracy of identifying anatomical of biliary tract than control group (Table 3, Fig. 5).

**Table 2:** Results of pretest and every 2-week test

Bile ducts with correct numbers and procedure time in group A (*n *= 10)									
	Left	Right	Right anterior	Right posterior	B IV	B VIII	Mean time (min)	Range	SD
Pretest	3	3	1	1	0	0	29	18–42	8
Examination 1	9	9	9	8	8	9	16	13–22	3
Examination 2	10	10	10	10	10	10	12	8–16	3
P1*	0.02	0.02	0.001	0.005	0.001	<0.001	0.001		
P2**	1	1	1	0.474	0.474	1	0.007		

*Difference between pretest and examination 1

**Difference between examination 1 and examination 2

**Table 3:** Results of posttest

Bile ducts and correct numbers during posttest						
	Left	Right	Right anterior	Right posterior	B IV	B VIII
Group A (*n *= 10)	10	10	10	9	8	10
Group B (*n *= 10)	4	4	3	2	1	2
*p*	0.011	0.011	0.003	0.005	0.005	0.001

## Discussion

For biliary tract diseases such as lithiasis, benign and malignant strictures, choledochoscopic examinations play an important role in the diagnosis and staging of such conditions. Due to the labyrinth-like anatomy of the biliary system and frequent anatomical variants [5], the inexperienced operator may be easily confused while performing choledochoscopic examinations. Thus, trainees usually require longer training periods to achieve familiarity with these techniques and to become comfortable with the complex anatomy of the biliary system.

3D reconstruction and 3DP have numerous applications in clinical medicine [6], but usually they are either used as visual models in the diagnosis of tumors, or as anatomical guidance tools for surgical procedures [7]. We propose a novel application of the 3D technology in the training of choledochoscopic techniques, which allows the trainee to gain direct feedback by continuously observing the model and manipulating the scope in anatomically realistic artificial biliary systems. Our participants reported favorable results in anatomy learning and skill acquisition, suggesting that 3DP has additional advantage over a simple 3D reconstruction image.

Experience in endoscopic ampullectomy training on a 3D printing model has been reported [8], but the biliary tract differs—from the gastrointestinal system as it is not a single-channel structure, but composed of a multitude of branches, adding to the difficulty of endoscopic manipulation in this system. Although the tracheal and bronchial structures resemble in a way the biliary tract, and the use of 3DP models in the training of rigid bronchoscopy has been reported, flexible endoscopes such as choledochoscopy are more difficult to control than rigid instruments, thus requiring longer training times to achieve the skills necessary to successfully manipulate a choledochoscopy. Therefore, trainees learning the techniques of choledochoscopy would benefit from realistic simulation models that closely resemble the actual clinical situation, similar to a model of endovascular training reported by Mafeld S *et al*, as examinations on real patients may not be readily available [9]. Our study suggests that technical training based on 3DP models can significantly improve the results of learning of such techniques, and this application of 3D technology has gained favorable feedback from both hepatobiliary surgeons and resident participants. We believe that this novel application has the potential to become an efficient learning tool in the training of flexible endoscopy in areas of complex anatomy such as the biliary tract that can eventually be included in the curriculum of residents training in hepatobiliary surgery.

There are certain limitations to the proposed 3DP training model and our study. First of all, the number of residents and models enrolled in this research is quite small, which means a large-scale trail is necessary in future. Secondly, biliary tract anatomical variations are common, and practice with a realistic model does not guarantee errorless manipulations of the choledochoscopy in real clinical situations. Thirdly, 3DP models are not capable of reproducing biological tissue elasticity due to the mechanical properties of the printing materials. Thus, the loss of normal texture and smoothness does not entirely reflect the mechanical properties of the bile ducts and may affect the tactile feedback provided to the trainee. Fourthly, since the models used for the pre- and posttests are different, technical familiarity with one 3DP model may not be readily transferred to another model; thus, the results of the posttests may not always accurately reflect the trainee’s skill level and learning progress. Another limitation is the fact that specific choledochoscopic examinations of B V–VII and B II–III were not carried out in the evaluations, due to the particularly difficult angles of the bile ducts entering these liver segments. Our results also showed that the correct identification rate of B IV and B VIII is relatively lower, suggesting that these segments may be exceptionally difficult to intubate and will require better training methods. Other options that may increase familiarity in difficult biliary tract navigation include augmented reality processes and electromagnetic navigation [10]. These techniques, combined with 3DP models, may eventually improve anatomical teaching and facilitate learning of choledochoscopy, leading to more efficient and accurate diagnosis and treatment for bile duct diseases. Limitations of our work include the small number of participants, and the fact that we do not have data on whether the trainees were able to consolidate their techniques on the long term. After first 2 weeks, trainees’ capacity progressed visibly; however, after second 2 weeks, it improved but indistinctively. We cannot draw the conclusion that 2 weeks is enough for training because improvement is not that obvious after first 2 weeks. Firstly, it is never enough to improve for patient’s safety. Secondly it may be bottleneck effect. Also, these junior resident trainees have not yet received training on real patients, thus the clinical learning curve is limited. Further studies with longer follow-up and more subjects may validate our findings.

## Conclusion

We have adopted the 3DP technology in the application of choledochoscopy training. Although a pilot study with a small number of participants and only two models, our results show that 3DP models may improve learning of such techniques and have the potential for considerable academic value. Evaluations of this strategy on a larger scale will validate our results, and allow for further technical development and novel applications in the area of choledochoscopy training, allowing trainees to exercise on anatomical accurate models with high clinical resemblance to real biliary systems.
